# Diversity of a wall-associated kinase gene in wild and cultivated barley

**DOI:** 10.1371/journal.pone.0218526

**Published:** 2019-06-27

**Authors:** Beata I. Czajkowska, Glynis Jones, Terence A. Brown

**Affiliations:** 1 School of Earth and Environmental Sciences, Manchester Institute of Biotechnology, University of Manchester, Manchester, United Kingdom; 2 Department of Archaeology, University of Sheffield, Northgate House, Sheffield, United Kingdom; Murdoch University, AUSTRALIA

## Abstract

Domestication of barley and other cereals was accompanied by an increase in seed size which has been ascribed to human selection, large seeds being preferred by early farmers or favoured by cultivation practices such as deep sowing. An alternative suggestion is that the increase in seed size was an indirect consequence of selection for plants with more vigorous growth. To begin to address the latter hypothesis we studied the diversity of *HvWAK1*, a wall-associated kinase gene involved in root proliferation, in 220 wild barley accessions and 200 domesticated landraces. A 3655-bp sequence comprising the gene and upstream region contained 69 single nucleotide polymorphisms (SNPs), one indel and four short tandem repeats. A network of 50 haplotypes revealed a complex evolutionary relationship, but with landraces largely restricted to two parts of the topology. SNPs in the *HvWAK1* coding region resulted in nonsynonymous substitutions at nine positions in the translation product, but none of these changes were predicted to have a significant effect on the protein structure. In contrast, the region upstream of the coding sequence contained five SNPs that were invariant in the domesticated population, fixation of these SNPs decreasing the likelihood that the upstream of a pair of TATA boxes and transcription start sites would be used to promote transcription of *HvWAK1*. The sequence diversity therefore suggests that the *cis*-regulatory region of *HvWAK1* might have been subject to selection during barley domestication. The extent of root proliferation has been linked with traits such as above-ground biomass, so selection for particular *cis*-regulatory variants of *HvWAK1* would be consistent with the hypothesis that seed size increases during domestication were the indirect consequence of selection for plants with increased growth vigour.

## Introduction

The transition from hunting-gathering to agriculture in the Fertile Crescent of southwest Asia was to a large extent based on the domestication of three cereal crops, einkorn wheat (*Triticum monococcum* L.), emmer wheat (*T*. *turgidum* L. subsp. *dicoccum* [Schrank ex Schübl.] Thell.) and barley (*Hordeum vulgare* L.) [1]. Although the pace and biogeography of cereal domestication is still disputed [2–4], the process resulted in crop plants that display phenotypic differences compared with their wild ancestors. These features are collectively referred to as the domestication syndrome [5,6], and include traits such as loss of the natural seed dispersal mechanisms and aids, insensitivity to environmental cues that inhibit germination and/or flowering, and an increase in size of individual seeds and/or the overall yield of grain per plant [6,7].

The domestication traits are assumed to have been selected by early cultivation practices, countering their low or zero adaptive advantage in wild populations [8], but the nature of the selective pressures that resulted in these evolutionary changes, and the role of conscious human agency in driving those changes, is the subject of intense debate [4, 9–13]. The increase in seed size that occurs during domestication provides an example. The size increase could have resulted from selection for large seeds, intended to provide greater food yield [14,15], or possibly because these were easier to handle [16]. Alternatively, cultivation practices might have involved burial of seeds to lower depths than encountered in the natural ecosystem, resulting in selection of seeds that had sufficient stored resource to support growth of the seedling during the increased period that would elapse before its emergence from the soil [14,17]. However, the first of these scenarios appears inconsistent with the relatively slow rate at which seed size increases over time, as indicated by the archaeobotanical record for at least some early farming sites [18], and the second is contradicted by experimental studies which have shown that, at least with the majority of legume species, the increased size of domesticated seeds compared with wild ones has no significant effect on the ability of seedlings to emerge after sowing at different depths [19]. An alternative explanation is that seed size increased as a pleiotropic effect of domestication, perhaps linked to the selection of plants with more vigorous growth. This hypothesis is supported by the observation that compared to their wild species, many domesticates, including southwest Asian cereals, have greater aboveground biomass with larger leaves and hence enhanced photosynthetic capabilities, as well as tall canopies and high leaf nitrogen contents [20–22]. Selection for growth vigour could also explain why the seeds of vegetables such as beet and carrot increase as a result of domestication, despite these crops being harvested for their roots or leaves rather than their seeds [23].

The preserved plant material at early agricultural sites mainly comprises seeds and, for cereals, intact spikelets and spikelet fragments such as glume bases [1,24], none of which provide information on the overall architecture or growth rate of the plants from which they were derived. The archaeobotanical record therefore does not allow us to address the hypothesis that early farmers selected for cereals with increased growth vigour. An alternative approach is to examine the sequence variability of genes influencing growth properties in living examples of the wild and domesticated versions of a crop, and then using the pattern of variability to assess whether particular haplotypes of those genes might have been selected during the domestication process. In barley, this approach has been successful in understanding selection for phenotypes such as flowering time [25] and seed shattering [26]. Using gene diversity to study selection for growth properties is more challenging because the genetic basis to growth vigour is a complex trait, but some individual genes that affect relevant phenotypes are known. An example in barley is *HvWAK1*, which codes for a member of the cell wall-associated kinase (WAK) group of receptor-like proteins. WAK proteins are located in plant cell walls and regulate disparate events including cell expansion and response to pathogens [27]. *HvWAK1* is expressed in germinating embryos and barley roots up to at least 28 days after planting, with no detectable expression in other tissues such as shoots, leaves, inflorescences and developing grain [28,29], and inactivation of *HvWAK1* results in a significant decrease in the rate of root elongation [28]. These expression and functional studies therefore indicate that the HvWAK1 protein plays a role in the control of root development, but is not directly involved in seed development. The extent of root proliferation has been linked with traits such as shoot growth rate, plant height and biomass production in rice and wheat [30,31], and hence can be used as a proxy for growth vigour. To begin to address the hypothesis that the increase in seed size that accompanied barley domestication was the indirect effect of selection by early farmers for plants with more vigorous growth, we examined the diversity of *HvWAK1* in an extensive range of georeferenced wild barley accessions and domesticated barley landraces.

## Materials and methods

### Barley accessions

The study material was 200 barley landraces (*H*. *vulgare* L.) and 220 wild barley accessions (*H*. *spontaneum* [K. Koch] Thell.) (S1 Table) obtained from the United States Department of Agriculture–Agricultural Research Service (USDA-ARS) Small Grains Collection (NSGC). Seeds were germinated at room temperature (c.22°C) in Petri dishes in hydroponic conditions. When coleoptiles emerged, the seeds were transferred to moist filter paper and seedlings grown until 21 days old. Fresh leaf material was then collected and DNA extracted using the ISOLATE II Plant DNA kit (Bioline).

### DNA sequencing

**Fig 1:**
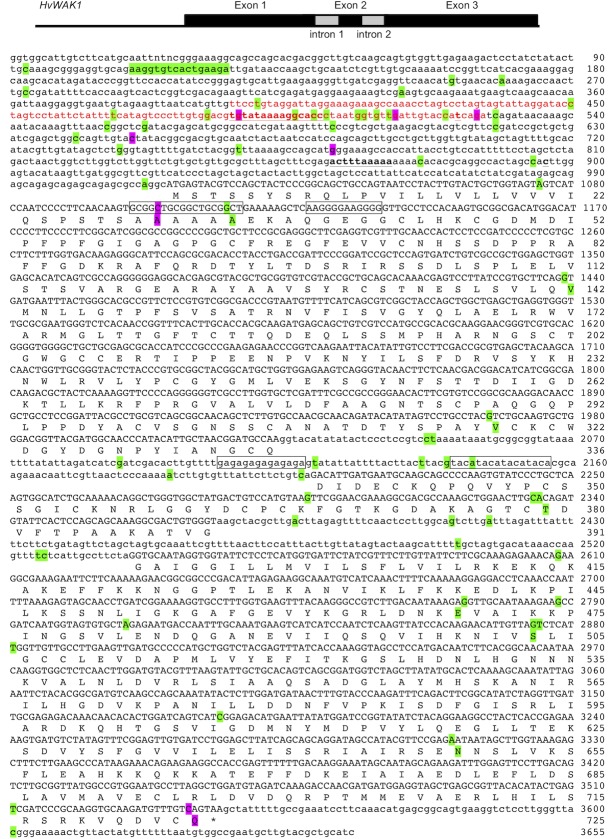
Consensus sequence of the *HvWAK1* gene and adjacent regions from 200 barley landraces and 220 wild accessions. The coding sequence is shown in upper case, and the sequence upstream of the initiation codon, the introns, and the sequence downstream of the termination codon in lower case. SNPs and the indel are highlighted in green, or purple for those seven SNPs that are invariant among landraces. The four STRs are boxed. In the upstream sequence, the region with closest similarity with the HORPIA–2 retrotransposon (see Results) is shown in red typeface, and the two predicted TATA boxes and their associated transcription start sites are shown in bold underlined.

A 3655 bp sequence comprising the entire *HvWAK1* gene (barleyGenes sequence MLOC_68187.1, EnsemblPlants locus HORVU5Hr1G087560|chr5H:578907334–57891028) along with the immediate upstream and downstream regions (Fig 1) was amplified as four overlapping fragments (amp1 primers: forward 5´–GGTGGCATTGTCTTCATGC–3´, reverse 5´–GATCCGGGAATCGGTCAG–3´, 1302 bp product, annealing temperature 68°C; amp2 primers: forward 5´–AGGCATGAGTACGTCCAGCTA–3´, reverse 5´–AATGTATGGGTTGCCATCGT–3´, 997 bp, 68°C; amp3 primers: forward 5´–GCGTGAGCTACAAGCACAAC–3´, reverse 5´–GCATCAACTTCAAGGCAACA–3´, 1238 bp, 68°C; amp4 primers: forward 5´–TGGTATTCTCCTCATGGTGATTC–3´, reverse 5´–GATGCAGCGTACAAGCATTC–3´, 1105 bp, 67°C). Polymerase chain reactions (PCRs) were carried out in a LightCycler480 (Roche) in 20 μl reaction volumes comprising 100 ng DNA extract, 1x SensiFAST SYBR No-ROX PCR master mix (Bioline), 100 nM forward primer, 100 nM reverse primer and PCR grade water. Cycling parameters were: 95°C for 5 min; followed by 35 cycles of 30 s at 95°C, 30 s at the annealing temperature, 60 s at 72°C. Product formation was assayed using the SYBR Green I/HRM Dye detection format (465 nm excitation, 510 nm emission), and melting data were obtained by first cooling the product to 55°C for 30 s and then heating to 99°C with five data acquisitions/°C. Melting peaks were obtained by plotting–(δF/δT) against temperature. PCR products were purified with the High Pure PCR Product Purification Kit (Roche) and sequenced using the BigDye Terminator v3.1 kit chemistry (Applied Biosystems). Standard sequencing reactions of 20 μl comprised 40 ng PCR product, 1x BigDye sequencing buffer, 0.125x BigDye reaction mix, 4 pmoles primer and UltraPure DNase/RNase-free distilled water. To avoid early signal loss when sequencing difficult regions (high GC/GT/G content, small hairpins or other secondary structures) a modified reaction of 20.05 μl was used, comprising 40 ng PCR product, 1x BigDye sequencing buffer, 0.125x BigDye v3.1 reaction mix, 0.0625x dGTP BigDye v3.0 reaction mix, 4 pmoles primer, 0.95 M betaine (Sigma), 5% (v/v) dimethyl sulfoxide (Sigma), UltraPure DNase/RNase-free distilled water. Cycling parameters were: 2 min at 96°C; 35 cycles of 40 s at 96°C, 15 s at 50°C, 4 min at 60°C; with products held at 4°C before purification (Agencourt CleanSEQ; Beckman Coulter) and reading of paired-end sequences by capillary electrophoresis in a 3730 DNA Analyser (Applied Biosystems).

### Data analysis

*HvWAK1* sequences for individual barley accessions were assembled from overlapping reads using Geneious version R10 (https://www.geneious.com, [32]). Multiple alignments were generated by the ClustalW, Muscle and Mafft programs and single nucleotide polymorphisms (SNPs) identified using the prediction software in Geneious at a maximum variant *p*-value of 10^−6^ and minimum sequence coverage of 250. Alignments of the *HvWAK1* consensus with other sequences was carried out with Clustal Omega [33] online at EMBL-EBI. Median joining haplotype networks were generated using Network 4 [34] and PopART [35]. Protein secondary structures were predicted using the garnier tool of EMBOSS [36], operated as a Geneious plug-in, and sequence features within the *cis*-regulatory region were identified using the transcription factor binding site function of PlantPAN 2.0 [37] and the TATA-TSS search function of TSSPlant [38], the latter online at Softberry (http://www.softberry.com/). Geographical distribution maps were plotted using ArcMap 10.2.1 of ArcGIS (ESRI. ArcGIS Desktop: Release 10. Redlands, CA: Environmental Systems Research Institute 2011).

## Results

Based on the published sequence of *HvWAK1* [28], we designed primers to amplify and sequence the gene in 200 landraces and 220 wild accessions of barley. The resulting consensus sequence, which differs in places from the published variant is 3655 bp, comprising 1016 bp upstream of the initiation codon, 2528 bp of coding sequence and introns, and 111 bp downsteam of the termination codon (Fig 1). The individual sequences displayed extensive variability (S2 Table) with 69 SNPs and one indel identified using the stringency parameters described in Materials and Methods. In addition, there are four short tandem repeats (STRs), two in the first exon of the coding sequence and two in the first intron.

**Fig 2:**
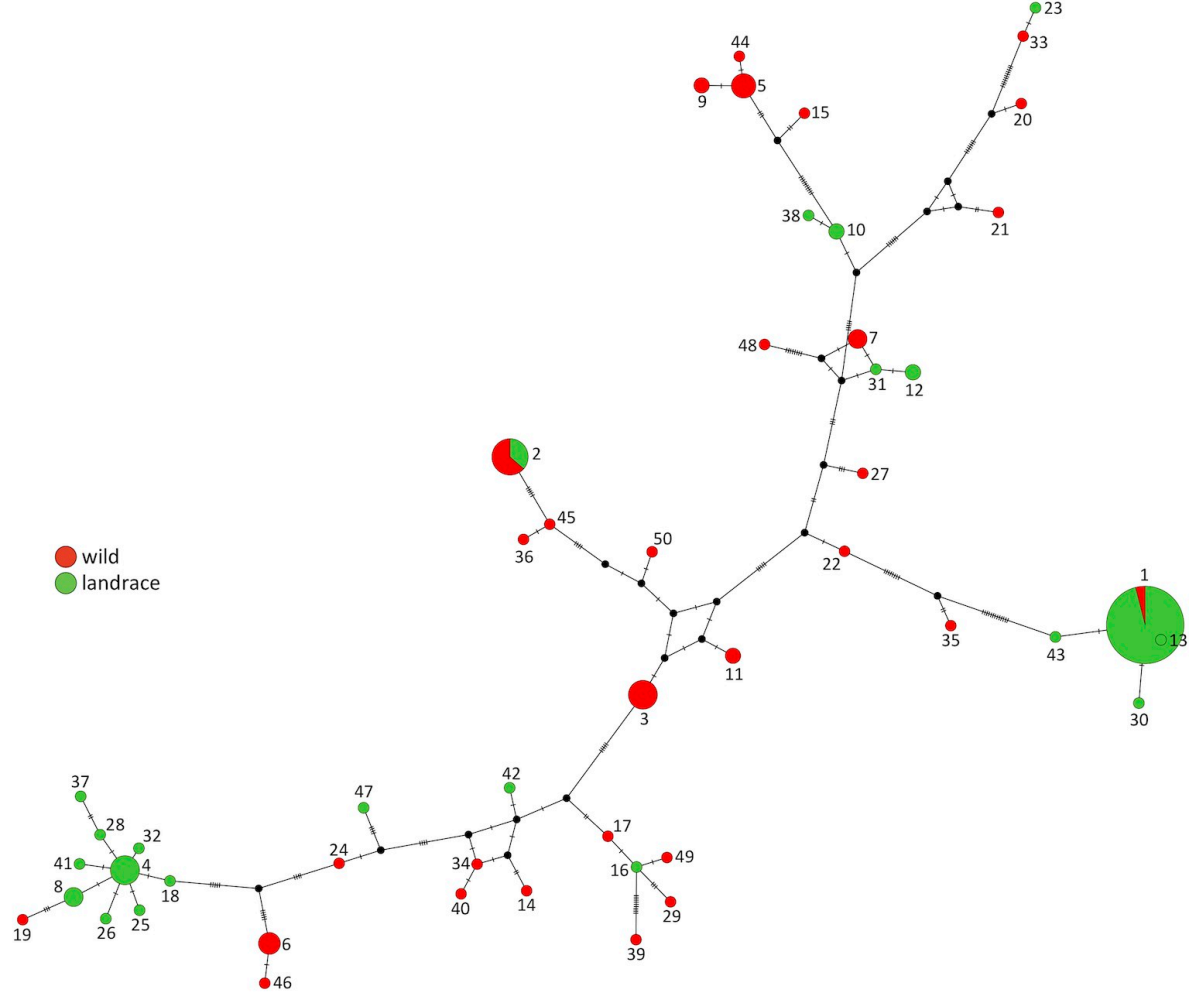
Network displaying the relationships between the 50 haplotypes of *HvWAK1*. Node sizes are proportional to numbers of accessions and empty nodes are shown as black dots. Short dashes on the edges indicate the number of point changes between pairs of nodes. The proportion of wild accessions and landraces for each haplotype are shown in red and green, respectively.

For 137 accessions (82 landraces and 55 wild barleys) complete sequences were obtained for all 69 SNPs, the indel and the four STRs, with no missing data. When variants at the SNPs and indel were taken into account, these sequences fell into 50 haplotypes (Table 1, S3 and S4 Tables). A single major haplotype included 48 landraces and 2 wild accessions, eleven other haplotypes had 2–12 members, and the remaining 38 were singletons comprising just one accession each. Inclusion of the STRs in the haplotype analysis did not result in significant changes: each of the 50 haplotypes was monoallelic for STRs 1, 2 and 4, and 47 haplotypes were also monoallelic for STR3. The exceptions were haplotypes 1, 2 and 5, each of which included two variants of the (GA)_n_ STR3 located within intron 1. Network analysis (Fig 2) revealed a complex evolutionary relationship between the 50 haplotypes, but with landraces largely restricted to two areas of the network, the first of these regions comprising major haplotype 1 and the singleton landrace haplotypes 13, 30 and 43, and the second made up of a starburst topology centered on haplotype 4 with haplotypes 8, 18, 19, 25, 26, 28, 32, 37, and 41 as satellites.

**Table 1:** *HvWAK1* haplotypes.

Haplotype	Number of accessions		
	Wild	Landraces	Total
**1**	2	48	50
**2**	8	4	12
**3**	7	0	7
**4**	0	7	7
**5**	5	0	5
**6**	4	0	4
**7**	3	0	3
**8**	0	3	3
**9**	2	0	2
**10**	0	2	2
**11**	2	0	2
**12**	0	2	2
**13–50**^a^	22	16	38

^a^Singleton haplotypes with one member each.

Nineteen of the SNPs are located within the coding sequence of the gene, nine of these SNPs resulting in amino acid changes in the predicted translation product, another nine giving synonymous codon changes, and one introducing a premature termination codon that shortens the predicted translation product by one amino acid (Table 2). The two STRs in the first exon of the coding sequence also result in amino acid sequence changes (S5 Table). The first of these STRs is (GCGGCT)_2–4_, specifying a series of 4–8 alanines, although the second of these alanines is valine in some accessions due to the presence of SNP35, and the fifth of can be proline due to SNP36. The second STR is (AAGGGG)_2–4_, which is translated into glutamine-glycine followed by 1–3 repeats of glutamic acid-glycine, followed by a single glycine.

**Table 2:** SNPs in the coding region of the *HvWAK1* gene.

SNP number	Nucleotide position	Amino acid position	Codon sequence		Amino acid identity	
			Consensus	Variant	Consensus	Variant
**SNP34**	1075	20	GTA	GTC	val	val
**SNP35**	1104	30	GCT	GTT	ala	val
**SNP36**	1115	34	GCT	CCT	ala	pro
**SNP37**	1439	142	GTG	CTG	val	leu
**SNP38**	1967	318	GTC	ATC	val	ile
**SNP49**	2298	366	AAG	AAA	lys	lys
**SNP50**	2334	378	TGC	TGT	cys	cys
**SNP51**	2335	379	ACA	TCA	thr	ser
**SNP58**	2608	414	CAG	CAA	gln	gln
**SNP59**	2773	469	GAG	GAT	glu	asp
**SNP60**	2788	474	AAG	AAA	lys	lys
**SNP61**	2809	481	CTA	CTC	leu	leu
**SNP62**	2874	503^a^	AGT	AAT	ser	asn
**SNP63**	2875	503^a^	AGT	AGG	ser	arg
**SNP64**	2881	505	ATT	ATC	ile	ile
**SNP65**	3184	606	ATC	ATT	ile	ile
**SNP66**	3311	649	AAT	CAT	asn	his
**SNP67**	3511	715	AGT	AGC	ser	ser
**SNP68**	3539	725	CAG	TAG	gln	stop

^a^When variants are present at both SNP62 and SNP63, amino acid position 503 is converted to AAG = lys.

Complete data for the nine nonsynonymous SNPs were available for 298 accessions (154 wild accessions and 144 landraces). The amino acid substitutions resulting from these SNPs combine to produce 28 protein variants (Table 3 and S6 Table), of which 16 are unique to the wild population, two are unique to landraces (these two variants comprising only three accessions) and the remaining ten are shared by wild accessions and landraces.

**Table 3:** Variants of the *HvWAK1* protein sequence.

Variant number	Amino acid sequence^a^	Number of accessions		
		Wild	Landraces	Total
**1**	AAVVTESNQ	50	26	76
**2**	A-VVTERHQ	4	69	73
**3**	AAVITDSNQ	4	27	31
**4**	AAVVSESNQ	17	–	17
**5**	A-VVTESHQ	12	4	16
**6**	AAVVTEKNQ	13	–	13
**7**	APVVTESNQ	9	1	10
**8**	AALVTEKN*	9	–	9
**9**	V-VVTENNQ	3	4	7
**10**	A-VVTESNQ	7	–	7
**11**	V-VITDSNQ	1	5	6
**12**	AALVTESNQ	3	3	6
**13**	AAVITESNQ	5	–	5
**14**	AAVVTDSNQ	3	–	3
**15**	A-VVTEKNQ	1	1	2
**16**	AAVVTESN*	2	–	2
**17**	AAVVTESNQ	–	2	2
**18**	A-VITDSNQ	1	1	2
**19**	V-VVTESNQ	2	–	2
**20**	A-LVTDSNQ	1	–	1
**21**	A-VVTDSNQ	1	–	1
**22**	AALITDSNQ	1	–	1
**23**	AALVTEKNQ	1	–	1
**24**	AAVITDNNQ	1	–	1
**25**	AAVITENNQ	1	–	1
**26**	APVVTEKNQ	1	–	1
**27**	APVVTERNQ	–	1	1
**28**	V-VITESNQ	1	–	1

^a^Amino acid identities at positions 30, 34, 142, 318, 379, 469, 503, 649 and 725, given in the IUPAC one-letter code.

* An asterisk indicates a stop codon at position 725.

The nonsynonymous amino acid changes include three that result in significant changes in chemical properties [39]. These are: the serine to arginine substitution at amino acid position 503 of the predicted translation product, caused by SNP63; the conversion of the same serine to lysine due to the presence of SNPs 62 and 63 in the codon specifying amino acid 503; and the asparagine to histidine change at position 649, caused by SNP66 (see Table 2). However, secondary structure predictions do not suggest that these or any other of the substitutions occurring in the coding sequence would have a significant impact on the structure and, by inference, function of the *HvWAK1* protein (S1 Fig). The three possible substitutions at amino acid position 503 (serine to asparagine, arginine or lysine) are predicted to affect the precise conformation of a short series of turns, but substitutions elsewhere in the protein either have no effect on the predicted secondary structure, or change the lengths of the predicted structural elements in minor ways.

Each of the 69 SNPs, as well as the indel and each of the four STRs, displays variation in the wild population (S7 Table). In contrast, seven of the SNPs are invariant in the landraces that were sequenced, each displaying the major allele present in wild plants. Two of the invariant SNPs are located in the coding sequence, with the consequence that all of the landraces have alanine at amino acid position 30, and none of the landraces have the premature termination codon. The other five invariant SNPs are numbers 10, 18, 20, 27 and 30, located upstream of the coding region at positions 487, 512, 525, 650 and 772, respectively. Two potential recognition sites for the TATA-binding protein were identified in this region by the promoter analysis function of PlantPAN2.0, and both of these sites were independently identified as TATA boxes by TSSPlant (see Fig 1). The first of these TATA boxes lies at positions 486–500, with a predicted transcription start site (TSS) at position 522, giving an mRNA with a 5´-untranslated region (5´-UTR) of 494 nucleotides. This TATA box and its TSS both lie within a region of the *HvWAK1* sequence that shows similarity with part of the 3´ long terminal repeat (3´-LTR) of the barley *copia*-like retrotransposon HORPIA–2 [40] (S2 Fig). The second TATA box is predicted at positions 862–871, with the TSS at position 896 and a 5´-UTR of 120 nucleotides. When the sequence containing the minor variants of SNPs 10, 18, 20, 27 and 30 (i.e. the sequence not found in landraces) is used as the query, the TSSPlant prediction tool assigns a higher score to the upstream TATA box (Table 4). Replacing the SNPs with their major variants (i.e. the sequence found in landraces), has no effect on the likelihood scores for the downstream TATA-TSS combination, but decreases the likelihood scores for the TATA box at positions 486–500 and the TSS at position 522. Examination of each SNP in turn reveals that SNPs 10 and 20 have the greatest impact: replacement of the major variant of SNP10 increases the score for the upstream TATA box to the same figure as obtained with minor versions of all five SNPs, and replacement of the major variant of SNP20 has a similar effect on the TSS. Altogether, 112 wild accessions have the minor variant at one or more of SNPs 10, 18, 20, 27 and 30 (S8 Table), these accessions distributed throughout the entire geographical range of wild barley (S3A Fig). These 111 accessions include 25 with the minor variant of SNP10, distributed in the southern Levant and southeast Turkey, with an outlier to the east (S3B Fig), and 50 with one of the minor variants of SNP20, which have a similar distribution (S3C Fig).

**Table 4:** Results of promoter analysis of the *HvWAK1* upstream region.

Sequence						TSSPlant likelihood scores			
Version	SNP10	SNP18	SNP20	SNP27	SNP30	TATA-box 486–500	TSS 522	TATA-box 862–871	TSS 896
**Minor variants**^a^	A	A	A	T	A	7.5504	1.9686	5.2759	1.9787
**Minor variants**^a^	A	A	C	T	A	7.5504	1.9712	5.2759	1.9787
**Major variants**	G	G	G	C	G	6.6702	1.8783	5.2759	1.9776
**Minor SNP10**	A	G	G	C	G	7.5504	1.8930	5.2759	1.9776
**Minor SNP18**	G	A	G	C	G	6.6702	1.8927	5.2759	1.9776
**Minor SNP20**^a^	G	G	A	C	G	6.6702	1.9588	5.2759	1.9776
**Minor SNP20**^a^	G	G	C	C	G	6.6702	1.9660	5.2759	1.9776
**Minor SNP27**	G	G	G	T	G	6.6702	1.8783	5.2759	1.9776
**Minor SNP30**	G	G	G	C	A	6.6702	1.8783	5.2759	1.9787

^a^SNP20 has two variant forms, A present in 44 wild accessions, and C in six accessions.

## Discussion

We sequenced the *HvWAK1* gene–coding for a wall-associated kinase that influences root proliferation–in a large set of barley landraces and wild accessions. The gene displayed extensive diversity with nineteen SNPs and two STRs in the coding sequence and an additional 50 SNPs, two STRs and an indel in the two introns and the regions immediately upstream and downstream of the coding sequence. Despite this complexity, the diversity was structured into 50 haplotypes that could be displayed as a median joining network with minimal character conflict (see Fig 2). Landraces were distributed non-randomly within this network: of the 82 landraces with complete data for every variable site, 51 were located at a peripheral position comprising haplotype 1 and the singleton haplotypes 13, 30 and 43, and another 17 landraces were located at a second peripheral position, in a starburst comprising haplotype 4 as the principal node and eight other landrace haplotypes as satellites. The events occurring during and/or after domestication have therefore resulted in some parts of the wild diversity *of HvWAK1* being lost and segments of the remaining diversity proliferating in the landrace population, consistent with the expectations of a domestication bottleneck [41] or the more gradual loss of diversity following domestication suggested by examination of time-series of ancient crop genomes [42,43].

The 19 SNPs in the coding region include nine that result in nonsynonymous codon changes (two of these changes in the same codon, meaning that there are ten nonsynonymous amino acid substitutions in total), and one that introduces a premature stop codon, one position upstream of the stop codon used in the majority of accessions. A total of 26 protein variants are present in the wild population, but the reduction in diversity that accompanied domestication means that only ten of these variants are seen in landraces. The majority (122/144) of the landraces belong to variants 1–3, which have dissimilar amino acid combinations at the nine nonsynonymous sites, with only four out of eight amino acid identities (alanine at position 30, valine at 142, threonine at 379 and glutamine at 725 –see Table 3). Those three amino acids are also present in fifteen other protein variants, nine of which are not seen in landraces, suggesting that the reduced landrace diversity is not due to selection for particular protein variants. Additional evidence against selection for protein variants is provided by predictions of the secondary structures that would form in the protein, this analysis (S1 Fig) indicating that the amino acid substitutions resulting from the nonsynonymous SNPs would not have a significant effect on the structures of the protein variants. Secondary structure prediction is of limited utility as it has only c.65% accuracy, and cannot reveal changes in longer range interactions within a protein, so the possibility remains that one or more of the amino acid substitutions has a significant effect on protein function, but the available information provides no evidence for this.

To gain further insight into possible functional differences that might arise as a result of the diversity of the *HvWAK1* sequence, we compared the frequencies of the major and minor variants of every SNP in the wild accessions and landraces (S7 Table). This analysis revealed that for seven SNPs the major variant is fixed in the landrace population. Five of these 'invariant' SNPs lie upstream of the *HvWAK1* initiation codon, in the *cis*-regulatory region predicted to contain two TATA boxes and their associated transcription start sites. The upstream of the two TATA-TSS combinations lies within a sequence segment that is highly similar to the 3´-LTR of the barley retrotransposon HORPIA-2, and may have been captured by the *HvWAK1* gene from an adjacent retrotransposon at some time in the past. Analysis of the region containing the TATA boxes and TSSs with the TSSPlant predictive software [37] suggests that fixation of SNPs 10 and 20 (at positions 487 and 525) decreases the likelihood of the upstream TATA box and TSS being used to promote transcription of *HvWAK1*. As well as the five invariant SNPs, several other SNPs in the *cis*-regulatory region have significantly different major/minor variant frequencies in landraces compared with the wild accessions. In particular, the minor variant of SNP11, located within the upstream TATA box, is present in only one landrace, and the minor variants of SNPs 13 and 17, which are between this TATA box and its TSS, are much more frequent in landraces compared with wild accessions. The latter is also the case for two additional SNPs, numbers 25 and 28, which are located between the two predicted TATA boxes.

Comparison of the sequence diversity of *HvWAK1* in wild barley accessions and landraces therefore suggests that the events during and/or after domestication might have resulted in selection of particular variants of the *cis*-regulatory region. The alternative, that fixation of the ‘invariant’ SNPs was due to stochastic effects resulting from the geographical distribution of the SNP variants in the wild population, is less likely, as wild accessions with the minor versions of the invariant SNPs are found throughout the Fertile Crescent, with the minor variants of SNPs 10 and 20, which according to the predictive software have the greatest impact on TATA-TSS usage, located in both the southern Levant and the Syria/southeast Turkey border, areas proposed as the sites of barley domestication [26]. It therefore seems less likely that the invariant SNPs became fixed as a result of chance sampling only of wild plants lacking the minor versions of these SNPs during the initial cultivation of barley.

The suggestion that the phenotypic features that characterize the domesticated version of a plant result not only from changes in protein structures but also changes in the expression patterns of key genes [44], has been confirmed by transcriptome studies of crops as diverse as maize [45–47], tomato [48,49], cotton [50,51], soybean [52] and carrot [53]. It now accepted that human selection during plant domestication acted on *cis*-regulatory regions as well as the coding sequences of genes [54]. The evidence that we present in this paper indicates that the *cis*-regulatory region of *HvWAK1* might have been subject to selection during the domestication of barley. Confirmation of this hypothesis would require functional studies that tested whether fixation of one or more of SNPs 10, 18, 20, 27 and 30 results in changes in the expression pattern of *HvWAK1* and/or can be associated with phenotypic changes such an alteration in the dynamics or extent of root proliferation. Such studies might be challenging due to the need to address the possibility that the functional effects of fixation of these SNPs might only be apparent under certain cultivation conditions.

As *HvWAK1* plays a role in root proliferation [28], selection for particular *cis*-regulatory variants of this gene would be consistent with the hypothesis that plants with increased growth vigour had a selective advantage under cultivation, possibly because they were able to out-compete plants with lower biomass and photosynthetic capability, and/or because farmers displayed a preference for larger and more vigorous plants. Our results therefore provide preliminary support for the proposal [19,23] that the increase in seed size that occurred during barley domestication was the indirect consequence of selection for increased growth vigour, rather than a direct consequence of the selection for large seeds.

## Supporting information

S1 Fig Secondary structure predictions for the barley *HvWAK1* protein. (A) The consensus amino acid sequence. (B) The amino acid sequence containing all variants, with asparagine at position 503. (C) Comparison of the predicted structures for the region surrounding each of the position 503 variants. Structural codes: pink barrel, α-helix; yellow arrow, β-strand; blue hooked arrow, turn; grey wavy line, coil.
(TIFF)

S2 Fig Alignment between the *HvWAK1* upstream sequence and the 3´-LTR of the barley retrotransposon HORPIA-2. Nucleotide identities are indicated by asterisks and the two predicted TATA boxes and their associated transcription start sites are highlighted in yellow. The *HvWAK1* sequence is numbered as in Fig 1. The HORPIA–2 sequence is taken from positions 35775–36099 of Genbank entry AH014393.2. Upstream and downstream of these positions in AH014393.2 there is no significant similarity with the *HvWAK1* sequence.
(TIFF)

S3 Fig Locations of the collection sites for wild accessions. (A) All wild accessions with the minor variant at one or more of SNPs 10, 18, 20, 27 and 30; (B) accessions with the minor variant at SNP10; (C) accessions with the minor variant at SNP20.
(TIFF)

S1 Table Barley landraces and wild accessions used in this study. (XLSX)

S2 Table SNPs and other variants in the *HvWAK1* sequence. (XLSX)

S3 Table Variant identities for all haplotypes. (XLSX)

S4 Table Haplotype memberships. (XLSX)

S5 Table STR variants in the *HvWAK1* coding sequence. (XLSX)

S6 Table Protein variant memberships. (XLSX)

S7 Table Allele frequencies at the variable positions in the *HvWAK1* sequence. (XLSX)

S8 Table Wild accessions with minor variants at SNPs 10, 18, 20, 27 or 30. (XLSX)
