# Supplementary figures and images for: Diversity of a wall-associated kinase gene in wild and cultivated barley
Source: PLoS One. 2019 Jun 27;14(6):e0218526. doi: 10.1371/journal.pone.0218526 (PMC6597065; doi:10.1371/journal.pone.0218526)

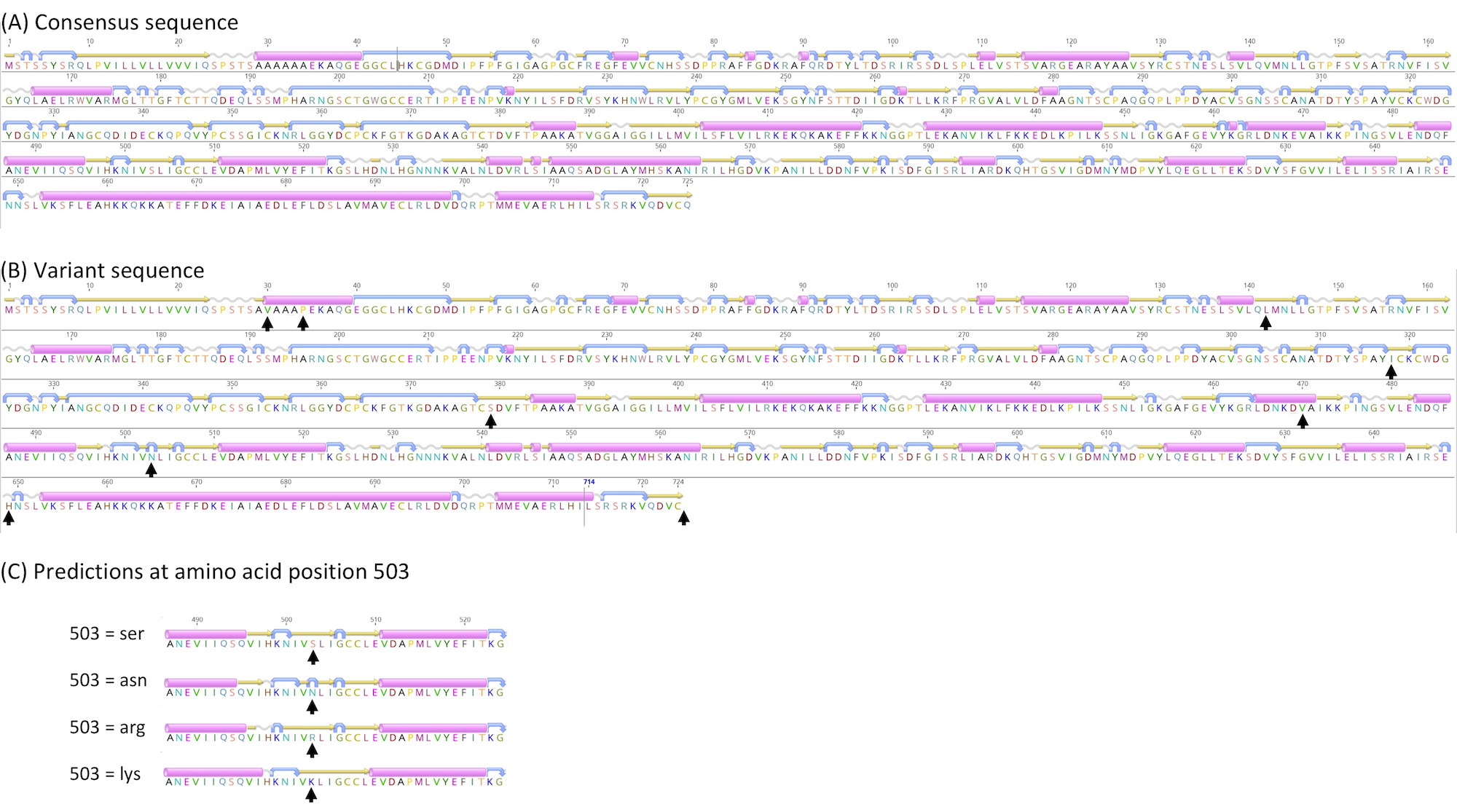

Supplement: S1 Fig — (A) The consensus amino acid sequence. (B) The amino acid sequence containing all variants, with asparagine at position 503. (C) Comparison of the predicted structures for the region surrounding each of the position 503 variants. Structural codes: pink barrel, α-helix; yellow arrow, β-strand; blue hooked arrow, turn; grey wavy line, coil. (TIFF) [file pone.0218526.s001.tiff]

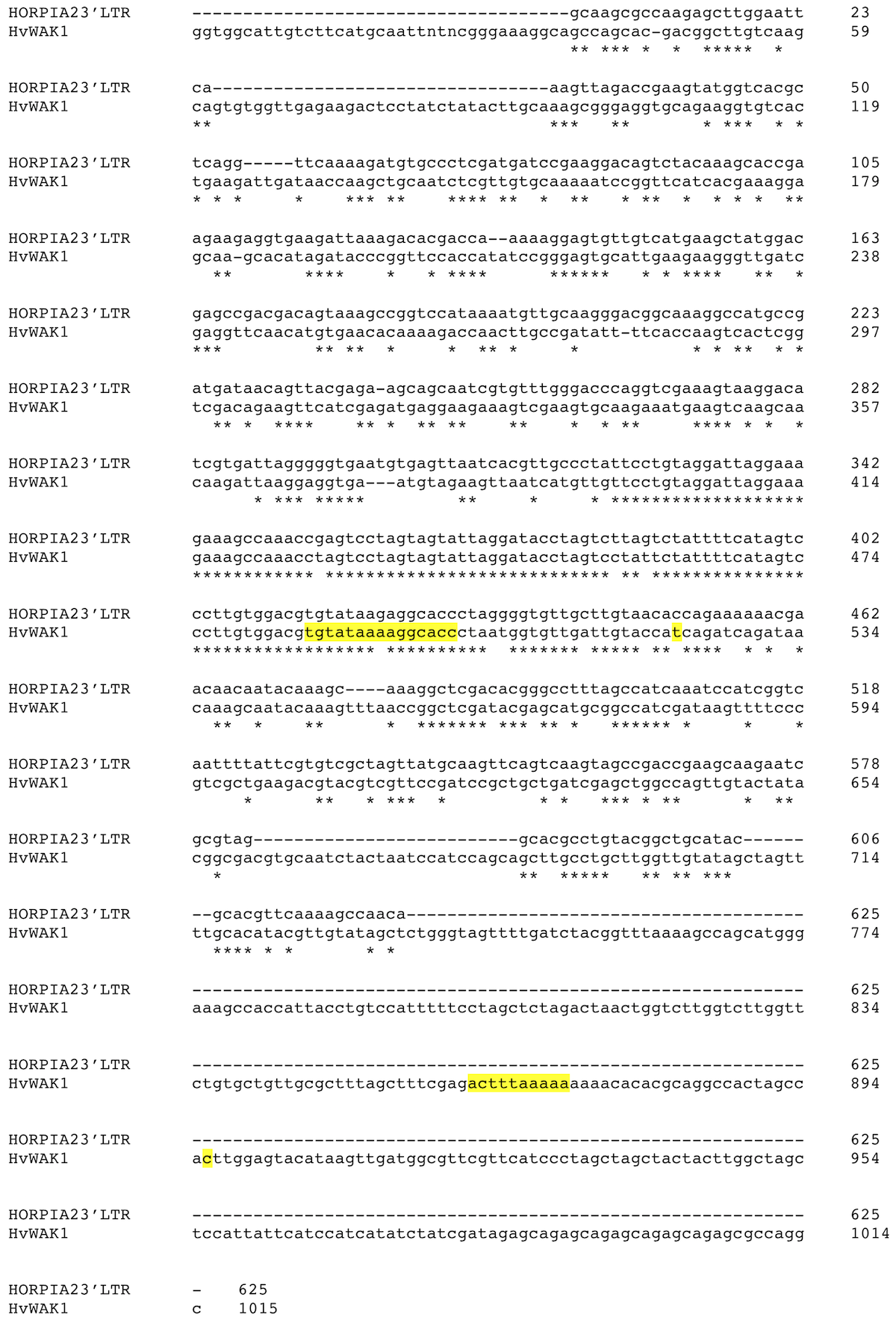

Supplement: S2 Fig — Nucleotide identities are indicated by asterisks and the two predicted TATA boxes and their associated transcription start sites are highlighted in yellow. The HvWAK1 sequence is numbered as in Fig 1. The HORPIA–2 sequence is taken from positions 35775–36099 of Genbank entry AH014393.2. Upstream and downstream of these positions in AH014393.2 there is no significant similarity with the HvWAK1 sequence. (TIFF) [file pone.0218526.s002.tiff]

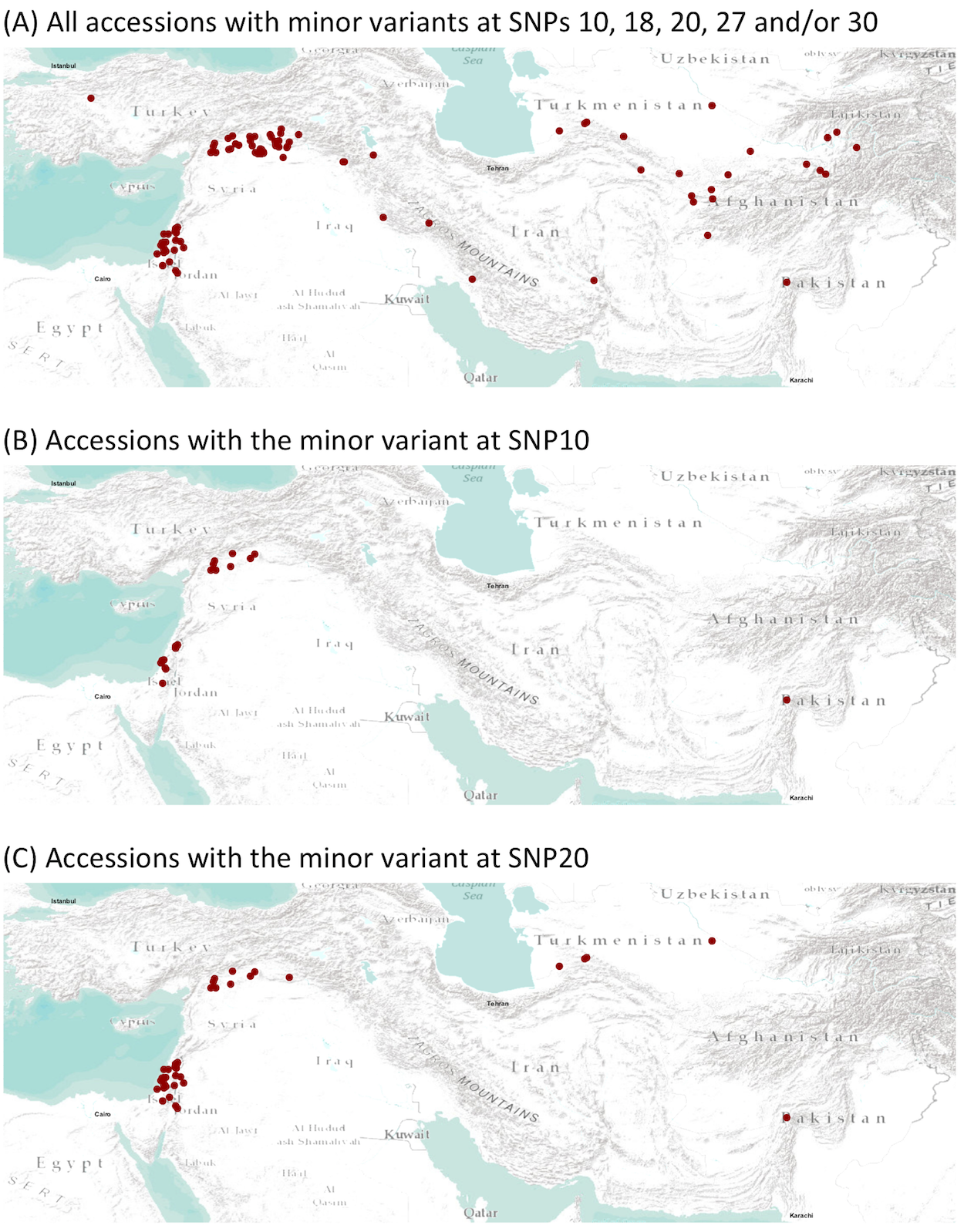

Supplement: S3 Fig — (A) All wild accessions with the minor variant at one or more of SNPs 10, 18, 20, 27 and 30; (B) accessions with the minor variant at SNP10; (C) accessions with the minor variant at SNP20. (TIFF) [file pone.0218526.s003.tiff]
